# Linked colour imaging benefits the endoscopic diagnosis of distal gastric diseases

**DOI:** 10.1038/s41598-017-05847-3

**Published:** 2017-07-17

**Authors:** Xiaotian Sun, Yiliang Bi, Tenghui Dong, Min Min, Wei Shen, Yang Xu, Yan Liu

**Affiliations:** 1 Department of Gastroenterology, the 307 Hospital of Academy of Military Medical Science, Beijing, 100071 China; 2 Department of Internal Medicine, Clinic of August First Film Studio, Beijing, 100161 China

## Abstract

Gastric diseases are common in China, and gastroduodenoscopy could provide accurate diagnoses. Our previous study verified that linked colour imaging (LCI) can improve endoscopic diagnostic accuracy. This study aimed for the first time to establish an LCI-based endoscopic model called colour-microstructure-vessel (CMV) criteria and validated its clinical feasibility for detecting distal gastric diseases manifested as red mucosal lesions under endoscopy in a cohort of 62 patients. Colour features were extracted from the endoscopic images and categorized into 3 types. Colour type 1 was a typical red; Colour type 2 was red ringed with purple and Colour type 3 was red with yellow in the centre and purple around the periphery, allowing for predicting chronic nonatrophic gastritis, chronic atrophic gastritis and gastric cancer. The sensitivity, specificity and Youden index of Colour type 3 with abnormal M or V for gastric cancer were 100.0%, 98.2% and 98.2%. The kappa values for intra-observer and inter-observer agreement for predicting the pathology were 0.834 and 0.791 for experienced endoscopists and 0.788 and 0.732 for endoscopy learners, and these values were comparable regardless of the experience of the endoscopists (P > 0.05). These findings support that the CMV criteria are a promising model for accurate endoscopic diagnosis.

## Introduction

Gastric diseases have a very high incidence in China and have been on the increase in recent years^1^. The clinical manifestations include upper abdominal pain, discomfort, distension and other signs or symptoms. Gastroduodenoscopy examination is the most useful diagnostic tool for multiple gastric diseases. Chronic gastritis (including chronic nonatrophic and atrophic gastritis) and gastric cancer are commonly observed in the distal stomach^2, 3^. Thus, it is of clinical significance to differentiate chronic nonatrophic gastritis, chronic atrophic gastritis and gastric cancer if a lesion is suspected in the distal stomach.

Along with significant advancements in the imaging technique, the endoscopic diagnosis efficacy has been greatly improved^4^. The clinical application of chromoendoscopy and magnified endoscopy in diagnosing gastrointestinal mucosal diseases has been validated in many studies. Linked colour imaging (LCI) is a newly developed endoscopic technique^5^ that integrates the colour post-processing function into the usage of short wavelength narrow-band laser light combined with white laser light based on the blue laser imaging (BLI)-bright mode. LCI could make the red area appear redder and the white area appear whiter, thus making it easy to detect any colour change of the mucosa; the colour observation of the mucosa under endoscopy could provide valuable evidence for an accurate diagnosis^6^. Our previous study reported that LCI could improve the endoscopic diagnosis accuracy. Additionally, the calculation of pixel brightness was identified as a promising quantifiable marker for evaluating the endoscopic images^7^, which supported that LCI could rapidly detect any colour changes during the endoscopic examination. Moreover, magnified endoscopic diagnosis using narrow-band imaging (NBI) and BLI has been established^8^. Thus, mucosal colour changes under the LCI mode can serve as an initial observation, while NBI and BLI can be applied for further confirmatory observations. In this study, we aimed to summarize and present new endoscopic diagnostic criteria called the colour-microstructure-vessel (CMV) criteria for differentiating the distal gastric diseases reported previously in the literature and from our experience. These criteria combine the rapid detection of the colour changes under the LCI mode with the traditional observation of microstructure and vessels to obtain an accurate diagnosis for gastric diseases, especially gastric cancer. The CMV criteria could further expand the current understanding of this new technique and improve the efficiency of endoscopic screening and diagnostic accuracy by specially emphasizing the mucosal colour changes.

## Results

### Demographic and clinical characteristics

A total of 62 patients with red mucosal lesions in the distal stomach that were identified using routine endoscopy were enrolled in the study (Table 1). White light endoscopy (WLE), LCI and BLI modes were used in all patients. The mean age was 50.1 ± 13 years, and 34 (54.8%) patients were male, and 28 (45.2%) patients were female. The main indications for upper gastroduodenoscopy examination included stomach pain (n = 21, 33.9%), abdominal distension (n = 31, 50.0%) and heartburn (n = 10, 16.1%) (Table 1).

**Table 1:** Demographic and clinical data.

	Patients (n = 62)
Age, mean ± SD, years	50.1 ± 13
Gender, n (%)	
Male	34 (54.8)
Female	28 (45.2)
Main indications, n (%)	
Stomach pain	21 (33.9)
Abdominal distension	31 (50.0)
Heartburn	10 (16.1)
CMV diagnosis, n (%)	
Chronic nonatrophic gastritis	19 (30.6)
Chronic atrophic gastritis	37 (59.7)
Gastric cancer	6 (9.7)
Pathological diagnosis, n (%)	
Chronic nonatrophic gastritis	14 (22.6)
Chronic atrophic gastritis	43 (69.4)
Adenocarcinoma	5 (8.1)

### CMV criteria for the distal stomach

**Figure 1:**
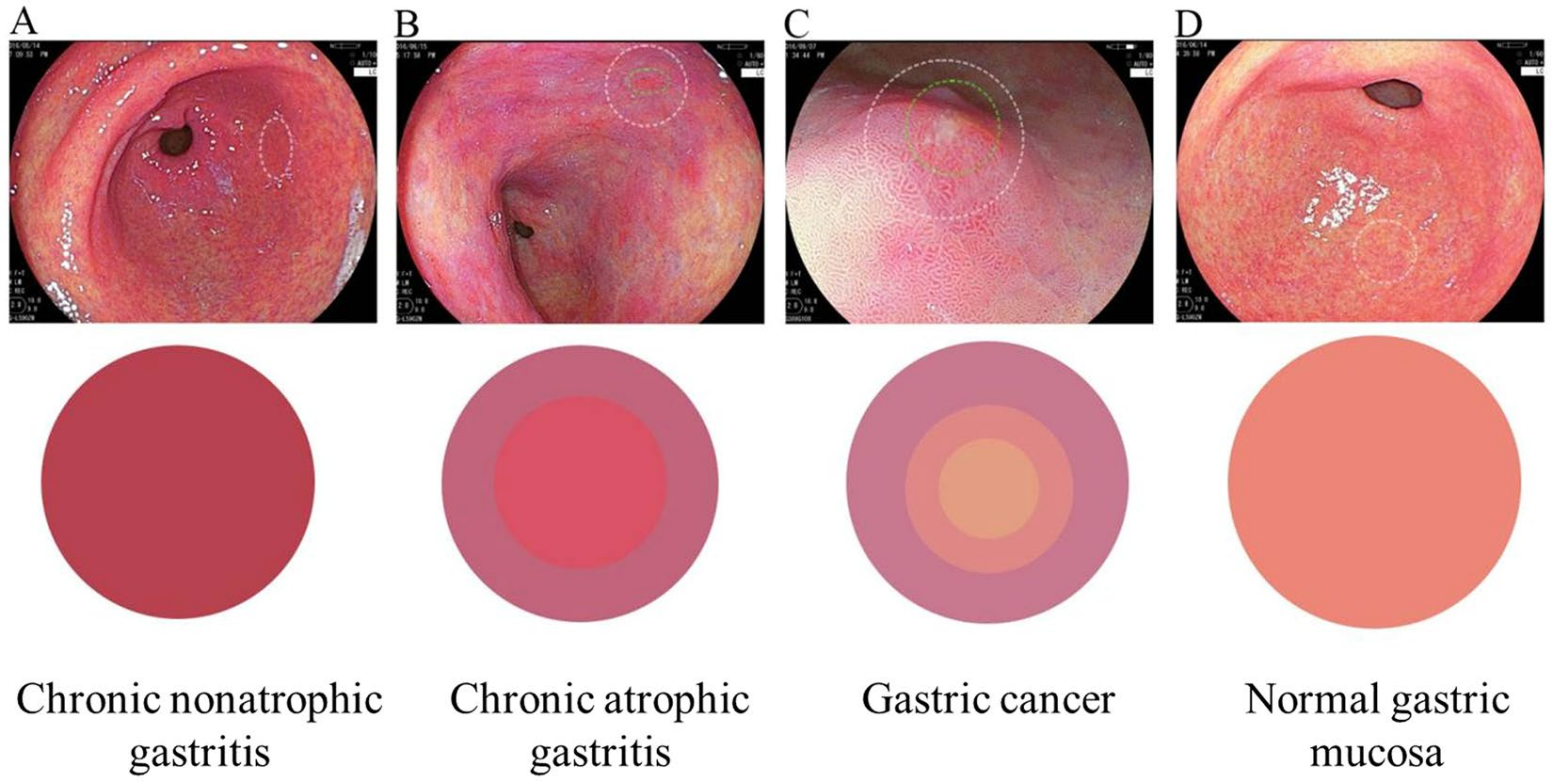
Colour types in the CMV criteria. (**A**) Colour type 1; (**B**) Colour type 2; (**C**) Colour type 3; (**D**) normal control.

Based on the CMV diagnostic criteria under the LCI mode proposed by our group, 19 (30.6%) patients with chronic nonatrophic gastritis, 37 (59.7%) patients with chronic atrophic gastritis and 6 (9.7%) patients with gastric cancer were diagnosed. The colour type was the most important feature of the CMV criteria, which was further analysed (Fig. 1) and could be used for the monitoring and rapid diagnosis of suspected lesions. The red mucosal lesions in the distal stomach were classified into 3 colour types, and the details are described in Table 2.

**Table 2:** Colour analysis and pixel brightness.

Colour types		R, mean ± SD	G, mean ± SD	B, mean ± SD	RGB model
1	Typical red	203.4 ± 27.4	105.9 ± 18.6	97.3 ± 15.7	Red
2	Red ringed with purple	206.2 ± 22.4	112.4 ± 20.3	130.9 ± 21.5	Purple
		212.5 ± 17.6	97.8 ± 18.2	107 ± 18.2	Red
3	Red with yellow in the centre and purple in the periphery	216.8 ± 15.8	140.6 ± 16.1	157.6 ± 14.9	Purple
		227.7 ± 7.6	119.5 ± 5.7	124.2 ± 14	Red
		227.8 ± 23.7	146.5 ± 40.4	127.2 ± 22.3	Yellow

Our previous study demonstrated that the calculation of pixel brightness for the red, green and blue colours from the typical endoscopic images could be used as a quantifiable biomarker for specific gastrointestinal mucosal lesions; therefore, the pixel brightness for the three colour types in the lesions were investigated first (Fig. 1). The pixel brightness for R, G and B in Colour type 1 was 203.4 ± 27.4, 105.9 ± 18.6 and 97.3 ± 15.7, respectively, indicating a typical red colour. The R, G and B pixel brightness in Colour type 2 was 212.5 ± 17.6, 97.8 ± 18.2 and 107 ± 18.2, respectively, in the central area and 206.2 ± 22.4, 112.4 ± 20.3 and 130.9 ± 21.5, respectively, in the surrounding area, indicating a red colour ringed with purple. In Colour type 3, a red colour (R 227.7 ± 7.6, G 119.5 ± 5.7, B 124.2 ± 14) with yellow (R 227.8 ± 23.7, G 146.5 ± 40.4, B 127.2 ± 22.3) in the centre and purple (R 216.8 ± 15.8, G 140.6 ± 16.1, B 157.6 ± 14.9) in the periphery was observed.

### CMV diagnosis could differentiate distal gastric diseases

**Figure 2:**
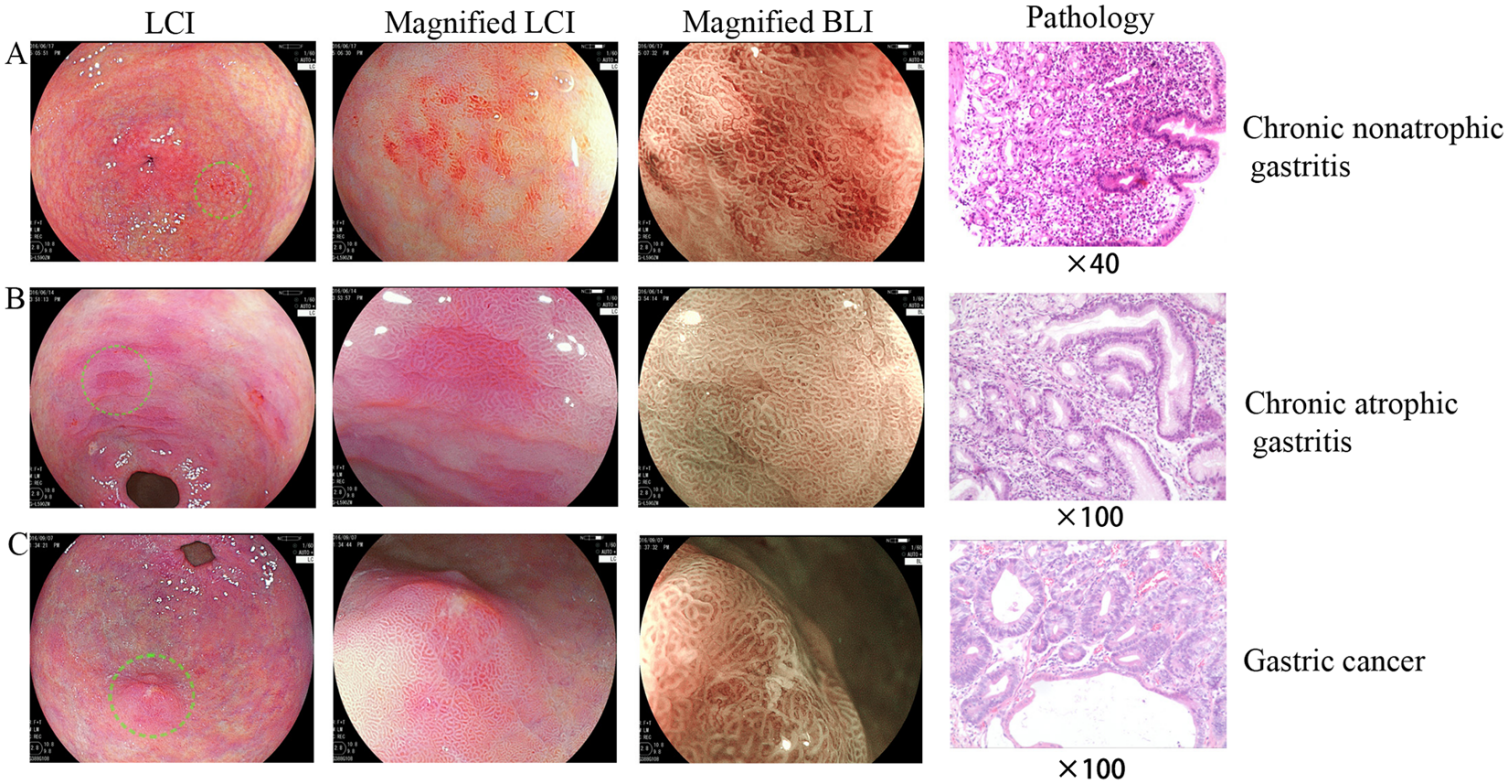
The CMV criteria were closely correlated with the pathological diagnosis. (**A**) chronic nonatrophic gastritis; (**B**) chronic atrophic gastritis; (**C**) gastric cancer.

To further deepen the role of the CMV criteria in clinical practice, we correlated the CMV diagnosis with the pathological diagnosis in our cohort of 62 patients (Fig. 2). The sensitivity, specificity and Youden index of the CMV diagnosis for gastric cancer was 100.0%, 98.2% and 98.2%, respectively (Table 3), which supports that the CMV criteria could provide an excellent diagnostic mode for detecting distal gastric diseases. Although the sensitivity, specificity and Youden index for the two main benign diseases in the distal stomach, namely, chronic nonatrophic gastritis (57.1%, 77.1%, 34.2%, respectively) and chronic atrophic gastritis (74.4%, 73.7%, 48.1%, respectively), were relatively low, these results could also support the conclusion that the CMV criteria had an advantage in differentiating benign lesions from gastric cancer in the distal stomach (Table 4, Supplementary Figure 1). However, one patient was misdiagnosed and was pathologically diagnosed with undifferentiated adenocarcinoma (ring cell carcinoma). It is suggested that our CMV criteria is applicable for moderately or well-differentiated tumours, which are manifested as white lesions in the background of intestinal metaplasia as purple lesions under LCI. This was also the reason why undifferentiated or poorly differentiated adenocarcinoma was most misdiagnosed, which was endoscopically observed as only white lesions.

**Table 3:** CMV diagnosis was highly correlated with pathological diagnosis (n = 62).

Patients, n (%)		Pathological diagnosis			Sensitivity, %	Specificity, %	Youden index, %
		Chronic nonatrophic gastritis (n = 14)	Chronic atrophic gastritis (n = 43)	Gastric cancer (n = 5)			
CMV diagnosis	Chronic nonatrophic gastritis (Colour type 1, n = 19)	8	11	0	57.1	77.1	34.2
	Pathological diagnosis (Colour type 2, n = 37)	5	32	0	74.4	73.7	48.1
	Gastric cancer (Colour type 3, with abnormal M or V, n = 6)	1	0	5	100.0	98.2	98.2

**Table 4:** Pathological findings of patients with gastric adenocarcinoma (n = 5).

Case	Size, cm	Lauren’s type	Location
No. 1	1.5 × 1.8	Intestinal type	Gastric antrum
No. 2	2.0 × 2.0	Intestinal type	Gastric corpus
No. 3	1.8 × 1.2	Intestinal type	Gastric angle
No. 4	1.5 × 1.8	Intestinal type	Gastric antrum
No. 5	1.5 × 1.0	Intestinal type	Gastric corpus

**Figure 3:**
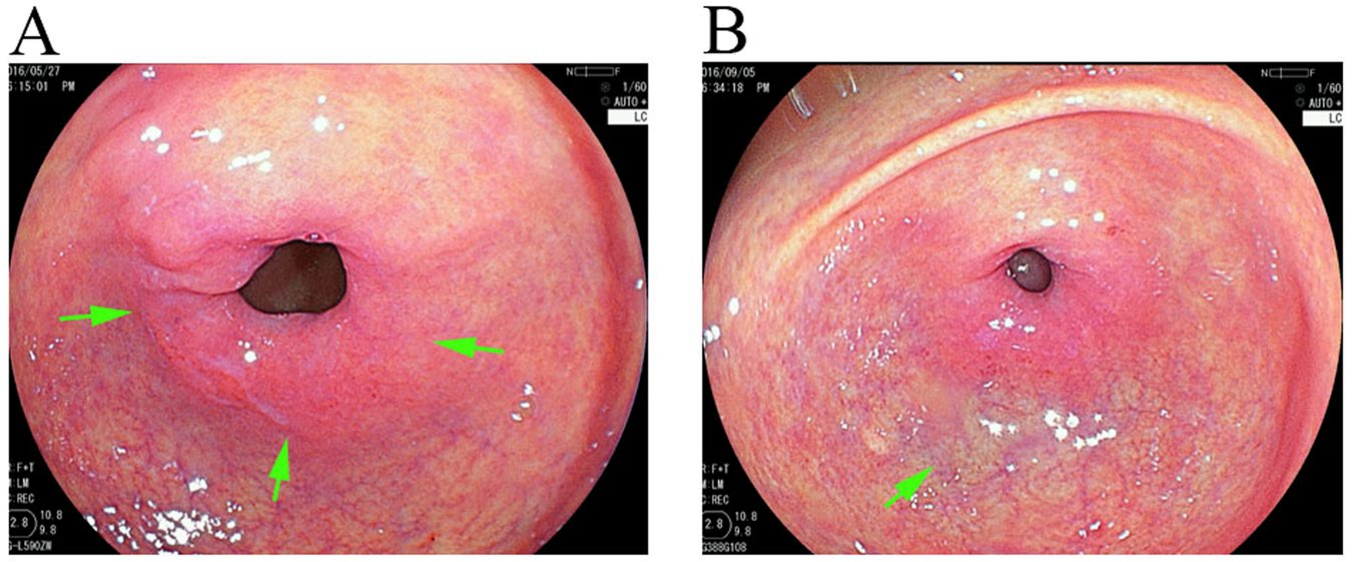
The purple pyloric ring (**A**) was manifested differently than the purple observed in the vessels (**B**).

We observed an obvious purple ring at a distance of 1 cm from the pylorus (Fig. 3). The pixel brightness value of R, G and B for the purple ring in the vicinity of the pylorus was 196.8 ± 19.0, 103.0 ± 18.3 and 113.3 ± 14.1, respectively. This purple colour of the pyloric ring was different from the purple colour observed in the vessels of the distal stomach, for which the R, G and B pixel brightness was 195.3 ± 7.9, 138.6 ± 22.8 and 140.6 ± 19.2, respectively. As a result, special attention should be focused on this type of colour change in the pylorus during endoscopic examination.

### Validation of CMV criteria

The kappa values for intra-observer and inter-observer agreement for predicting the pathology were 0.834 (95% confidence interval [CI] 0.723–0.891) and 0.791 (95% CI 0.714–0.822), respectively, in the experienced endoscopists group and 0.788 (95% CI 0.698–0.802) and 0.732 (95% CI 0.685–0.799), respectively, in the endoscopy learners group (Table 5). Furthermore, the kappa values for intra-observer and inter-observer reproduction in the experienced endoscopists group and the endoscopy learners group were comparable (P > 0.05), which indicates that the CMV criteria could be an easy and convenient tool for diagnosing distal gastric diseases regardless of the endoscopist experience.

**Table 5:** Intra-observer and inter-observer agreement.

κ value (95% CI) for predicting the pathology	Experienced endoscopists group	Endoscopy learners group
Intra-observer agreement	0.834 (0.723–0.891)	0.788 (0.698–0.802)
Inter-observer agreement	0.791 (0.714–0.822)	0.732 (0.685–0.799)

**Figure 4:**
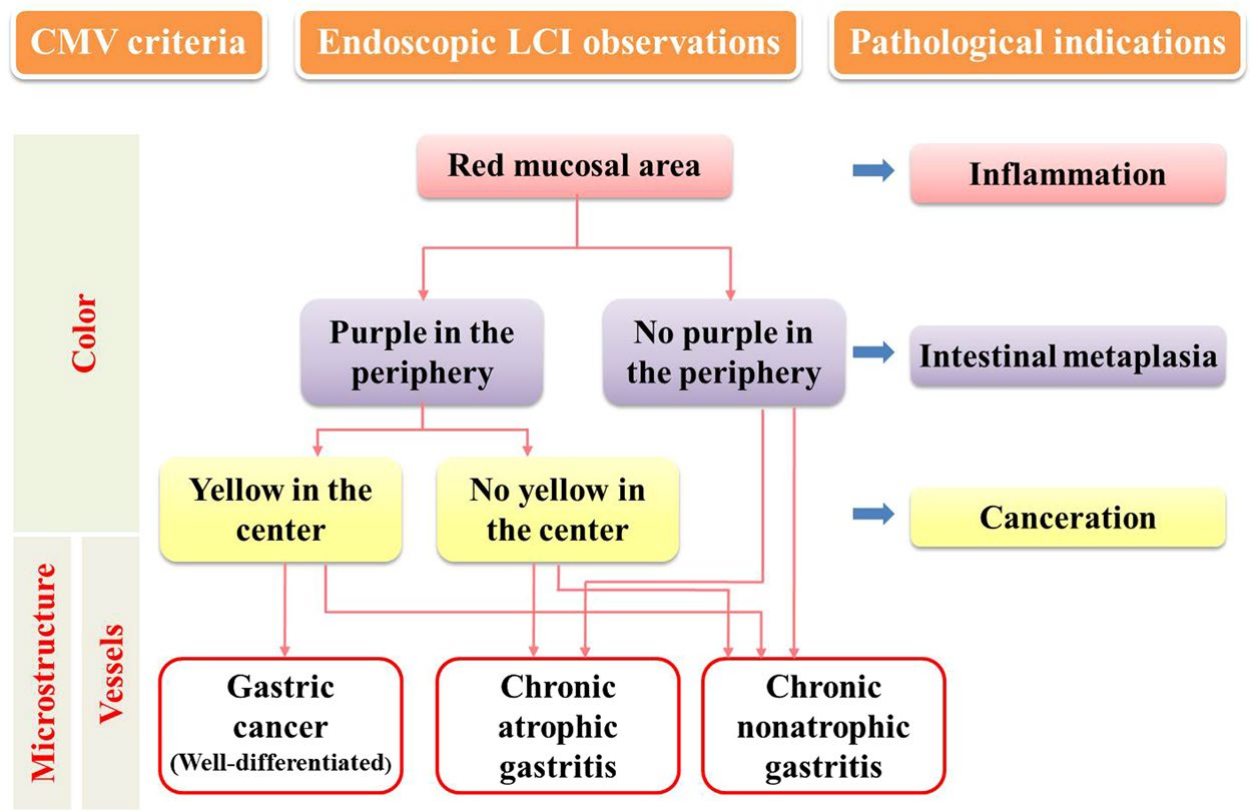
Diagram for the CMV criteria under LCI mode and the pathological indications.

The diagram for the CMV criteria is illustrated in Fig. 4. First, the LCI mode was used to observe the gastrointestinal mucosa and detect any red area that was a suspected lesion. Then, magnified LCI observation was applied to determine the colour type of the suspected lesion. The detection of the microstructure and vessels could help make a diagnosis and facilitate the targeted biopsy.

## Discussion

Gastric diseases are quite common in the mainland of China and are the most common indications for gastroduodenoscopy examination and endoscopic diagnosis in clinical practice. In recent decades, several new endoscopic imaging techniques have been developed^9–11^ that could either facilitate the endoscopic diagnosis or simplify the procedures^12–15^. LCI is a new endoscopic technique and is characterized by the capability of making the white mucosal area appear whiter and the red mucosal area appear redder, which makes it easier for the endoscopists to identify the mucosal abnormality^5^. Currently, few studies on the application of LCI have been conducted, and no LCI-based endoscopic diagnostic criteria have ever been proposed^16–18^. Thus, in this study, for the first time, we propose new endoscopic CMV criteria for differentiating distal gastric diseases based on our previous investigations involving the LCI technique and then validated the clinical feasibility and effectiveness. These findings may even benefit the diagnosis of surveillance endoscopy in such patients with chronic gastritis and premalignant conditions.

In the distal stomach, chronic atrophic gastritis, chronic nonatrophic gastritis and gastric cancer are commonly observed, and other types of diseases are rare, which may be explained by the special histology in this mucosal area. This is also the reason why we tried to first establish the CMV criteria for distal gastric diseases. In this study, the red mucosal lesions that were detected in the distal stomach via white endoscopy were focused; the majority of mucosal abnormalities were observed as red areas via endoscopy, especially inflammation and cancer^19^.

Analysis of the endoscopic images could provide direct evidence for diagnosing certain gastrointestinal diseases^20^. The establishment of the CMV criteria was aimed at determining 3 types of diseases according to the endoscopic observation of the colour, microstructure and vessels of the mucosa. The colour types were categorized from all the LCI images of the distal stomach in our computerized database. There were mainly 3 colour types including typical red (Colour type 1), red ringed with purple (Colour type 2) and red with yellow in the centre and purple in the periphery (Colour type 3). These types of colour changes may be explained by the pathology of the mucosal lesions^21, 22^, which is evidenced by the close correlation between the CMV diagnosis and the pathological diagnosis. Intestinal metaplasia was observed as purple under LCI, inflammation was observed as red, and the normal mucosa or tumour was observed as yellow. Chronic nonatrophic gastritis is a type of nonspecific inflammation in the distal stomach, which manifests as typical red under LCI. Chronic atrophic gastritis is closely associated with intestinal metaplasia and is consequently observed as red ringed with purple under LCI. The pathological changes of gastric cancer are complicated, and the colour changes are accordingly intricate. The non-malignant epithelial layer that covered the malignant glands was confirmed in one patient, and LCI had advantages of detecting the minor colour changes of the mucosa over BLI, which still needs to be further examined. The RGB pixel brightness values for each colour types were analysed and highly consistent with the described colour changes. In addition, the intra-observer and inter-observer reproduction assay for the CMV criteria showed that the CMV criteria for distal gastric diseases had good reliability for both experienced endoscopists and endoscopy learners. Anagnostopoulos GK *et al*. proposed a magnified endoscopy diagnosis for gastric body diseases, and the kappa values for inter-observer and intra-observer agreement in predicting normal gastric mucosa, chronic atrophic gastritis and gastric atrophy were 0.864 (95%CI 0.800–0.928) and 0.913 (95%CI 0.890–0.936), respectively^23^, which was comparable with our CMV criteria for diagnosing distal gastric diseases.

Taken together, the LCI technique is a feasible and effective method for observing mucosal colour changes, and the CMV criteria could be introduced as a new promising endoscopic diagnostic model for multiple diseases. However, our study only evaluated the CMV criteria for the differentiation of distal gastric diseases in a small cohort, and the CMV criteria for other gastrointestinal mucosal diseases were not analysed. A multicentre large scale clinical trial will be conducted in the future, at which time this criteria will be optimized and updated.

## Patients and Methods

### Patients

Consecutive patients who underwent LCI gastroduodenoscopy from May 1, 2016 to August 31, 2016 were retrieved from the computerized database in our endoscopy centre. Patients who had red flat mucosal lesions in the distal stomach under endoscopy were selected for this study. The demographic and clinical data were collected and analysed.

This study was approved by the Ethics Committee of the 307 Hospital of the Academy of Military Medical Science, and all the patients gave written informed consent. All methods were performed in accordance with the relevant guidelines and regulations.

### Endoscopic procedures

The endoscopic examinations were performed by the same experienced endoscopist using the EC-L590ZW endoscope with the LASEREO system (FUJIFILM Co., Tokyo, Japan). The main indications included upper abdominal discomfort, pain and distension. No anaesthesia was needed for any of the patients. The lesions and surrounding mucosa in the distal stomach were observed using 3 different modes (WLE, LCI and BLI), and the typical endoscopic images for each mode were saved. If patients had more than 1 lesion, all the lesions were recorded for further analysis.

We first identified the suspected lesion, which was abnormally red under WLE, and then used the LCI mode to observe the typical colour change of the lesion and the surrounding mucosa. Subsequently, the moderately magnified BLI mode was applied to detect the microstructure and vessels of the mucosa. The typical microstructure abnormality observed was irregularity or loss of the round pits. Loss of the normal subepithelial capillary network and irregular arrangement of the collecting venules were considered to be vascular abnormalities^23^. Biopsy was performed for each lesion and sent for pathological examination, which was the gold standard for diagnosis.

### Colour analysis of endoscopic images

Typical endoscopic images for the lesions were analysed using MatLab software (USA), and the pixel brightness of the red, green and blue (RGB) colours for the targeted areas was calculated^7, 24^. The distance to the mucosa was estimated using the top (approximately 2 mm) of a transparent cap (DH-11GZ, FUJIFILM Co., Tokyo, Japan) for comparison when taking images. The standard colour was synthesized based on the value of the RGB pixel brightness. The lesions were classified into 3 main colour types as follows: 1) Colour type 1, typical red; 2) Colour type 2, red ringed with purple; and 3) Colour type 3, red with yellow in the centre and purple in the periphery.

### Intra-observer and inter-observer reproducibility

In the second part of this study, one colour image under LCI, one microstructure image and one vessel image under BLI were selected for each lesion as a set from all the recorded endoscopic images. A total of 150 endoscopic image sets were chosen. Three experienced endoscopists and 3 endoscopy learners who were blinded to the histological analysis were invited to provide diagnosis for the selected images based on the CMV criteria that we described previously. The answers were recorded. Three weeks later, all the endoscopists were invited for a repeat evaluation of the images.

### Statistical analyses

All the statistical analyses were conducted using SPSS 17.0 software. The continuous and categorical data were presented as the means ± standard deviation (SD) and percentage (%). The chi-square test was used to compare the consistency of the endoscopic diagnosis for distal gastric diseases based on the CMV criteria with pathological diagnosis, and the sensitivity and specificity of the CMV criteria were calculated. The kappa values for the intra-observer and inter-observer agreement were also evaluated. A two-tailed P-value less than 0.05 was considered to be statistically significant.

## Electronic supplementary material

Supplementary info
