# Supplementary material for: Linked colour imaging benefits the endoscopic diagnosis of distal gastric diseases
Source: Sci Rep. 2017 Jul 17;7:5638. doi: 10.1038/s41598-017-05847-3 (PMC5514041; doi:10.1038/s41598-017-05847-3)

Linked colour imaging benefits the endoscopic diagnosis of distal gastric diseases

Xiaotian Sun^1,2*^, Yiliang Bi^1*^, Tenghui Dong^1*^, Min Min^1^, Wei Shen^1^, Yang Xu^1^, Yan Liu^1^

^1^Department of Gastroenterology, the 307 Hospital of Academy of Military Medical Science, Beijing 100071, China; ^2^Department of Internal Medicine, Clinic of August First Film Studio, Beijing 100161, China

* These authors contributed equally to this work.

Correspondence author: Yan Liu

Department of Gastroenterology, the 307 Hospital of Academy of Military Medical Science, 8 East Street, Fengtai District, Beijing 100071, China

Telephone/Fax: 86-010-66927473; Email: 13911798288@163.com.

Supplementary figure 1. Endoscopic images and pathological diagnosis of 4 cases with gastric cancer (A-D).


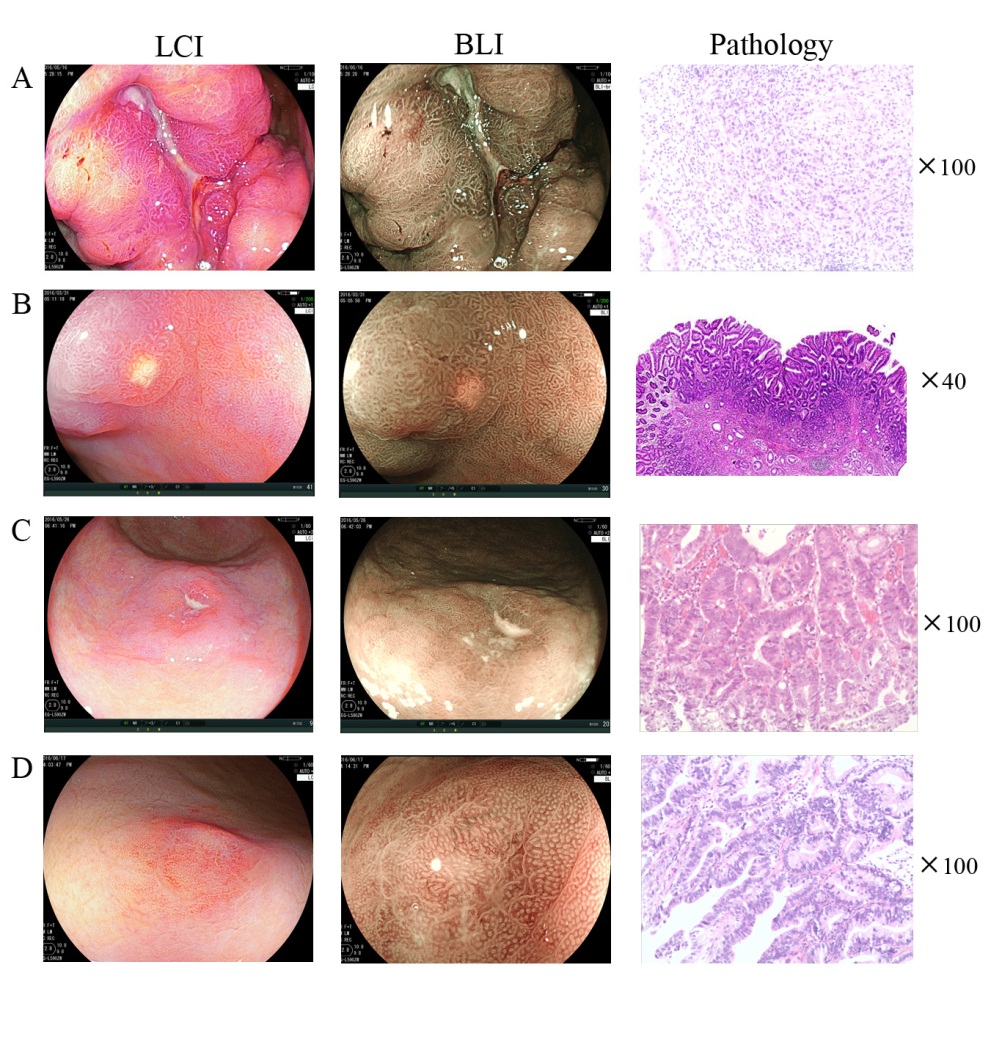

Supplement: Supplementary file 1 — Supplementary info [file 41598_2017_5847_MOESM1_ESM.doc]
